# Effects of sevoflurane anesthesia and abdominal surgery on the systemic metabolome: a prospective observational study

**DOI:** 10.1186/s12871-021-01301-0

**Published:** 2021-03-17

**Authors:** Yiyong Wei, Donghang Zhang, Jin Liu, Mengchan Ou, Peng Liang, Yunxia Zuo, Cheng Zhou

**Affiliations:** 1 Laboratory of Anesthesia & Critical Care Medicine, Translational Neuroscience Center, West China Hospital of Sichuan University, 37# Guoxue Xiang, Chengdu, 610041 Sichuan China; 2 Department of Anesthesiology, West China Hospital of Sichuan University, 37# Guoxue Xiang, Chengdu, 610041 Sichuan China

**Keywords:** Metabonomics, Sevoflurane, Observational study, Abdominal surgery

## Abstract

**Background:**

Metabolic status can be impacted by general anesthesia and surgery. However, the exact effects of general anesthesia and surgery on systemic metabolome remain unclear, which might contribute to postoperative outcomes.

**Methods:**

Five hundred patients who underwent abdominal surgery were included. General anesthesia was mainly maintained with sevoflurane. The end-tidal sevoflurane concentration (ET_sevo_) was adjusted to maintain BIS (Bispectral index) value between 40 and 60. The mean ET_sevo_ from 20 min after endotracheal intubation to 2 h after the beginning of surgery was calculated for each patient. The patients were further divided into low ET_sevo_ group (mean − SD) and high ET_sevo_ group (mean + SD) to investigate the possible metabolic changes relevant to the amount of sevoflurane exposure.

**Results:**

The mean ET_sevo_ of the 500 patients was 1.60% ± 0.34%. Patients with low ET_sevo_ (*n* = 55) and high ET_sevo_ (*n* = 59) were selected for metabolomic analysis (1.06% ± 0.13% vs. 2.17% ± 0.16%, *P* < 0.001). Sevoflurane and abdominal surgery disturbed the tricarboxylic acid cycle as identified by increased citrate and cis-aconitate levels and impacted glycometabolism as identified by increased sucrose and D-glucose levels in these 114 patients. Glutamate metabolism was also impacted by sevoflurane and abdominal surgery in all the patients. In the patients with high ET_sevo_, levels of L-glutamine, pyroglutamic acid, sphinganine and L-selenocysteine after sevoflurane anesthesia and abdominal surgery were significantly higher than those of the patients with low ET_sevo_, suggesting that these metabolic changes might be relevant to the amount of sevoflurane exposure.

**Conclusions:**

Sevoflurane anesthesia and abdominal surgery can impact principal metabolic pathways in clinical patients including tricarboxylic acid cycle, glycometabolism and glutamate metabolism. This study may provide a resource data for future studies about metabolism relevant to general anaesthesia and surgeries.

**Trial registration:**

www.chictr.org.cn. identifier: ChiCTR1800014327.

**Supplementary Information:**

The online version contains supplementary material available at 10.1186/s12871-021-01301-0.

## Introduction

Although volatile anesthetics have been used in clinical setting for more than 170 years, how volatile anesthetics impact physiological status is still elusive [1]. It is widely known that general anesthetics can impact some important processes of metabolism [2, 3] and metabolic connectivity [4]. For example, general anesthesia induced by sevoflurane can change metabolic activity in the central nervous system detected by functional neuroimaging [5]. Sevoflurane inhibits insulin secretion in rodents [2, 6] and general anesthesia with sevoflurane is associated with hyperglycaemia in human [7]. However, the exact modulation of general anesthetics on systemic metabolome in human has not been clearly illustrated. Understanding the exact effects of volatile anesthetics on systemic metabolism is important for perioperative management of patients, because it can be involved in postoperative recovery [8], postoperative cognitive function [9] and infection [10].

Metabolic status may impact sensitivity of general anesthetics in turn. Previous study indicates that sensitivity to general anesthetics is associated with metabolic function and/or status of the central nervous system. Impaired metabolic status caused by mitochondrial dysfunction, can alter the effects of volatile anesthetics on neural functions including disrupting excitatory neurotransmitter dynamics [11] and activity of thalamocortical circuit [12]. Isoflurane at clinically relevant concentrations can inhibit mitochondrial complex I, and function of mitochondrial complex I in excitatory neurons is relevant to the sensitivity of volatile anesthetics in vivo in mice [11]. The *C. elegans* lacking mitochondrial complex I subunit NDUFS4 is remarkably hypersensitive to isoflurane [13]. Oxidative phosphorylation-induced complex I dysfunction can also alter sensitivity to volatile anesthetics [13]. However, whether volatile anesthetics dose-dependently modulate metabolic function in humans is unknown.

In the present study, we hypothesized that general anesthesia and abdominal surgery can impact principal metabolic pathways in humans. Here we conducted a clinical observation to investigate the exact effects of sevoflurane and abdominal surgery on systemic metabolism for the first time.

## Methods

### Participants

The observational study was approved by the Ethical Committee of West China Hospital of Sichuan University on May 19, 2017 (Approval No. 78) and was registered prior to patient enrolment with the Chinese Clinical Trial Registry (ChiCTR1800014327, principal investigator, Yunxia Zuo) on Jan 6, 2018. All selected patients were aged 18–65 years with ASA physical status 1–2, and they underwent abdominal surgery with a duration exceeding 2 h between January 2018 and September 2018 at West China Hospital of Sichuan University. Written informed consent was obtained from all patients. The exclusion criteria were as follows: allergic to volatile anesthetics, cerebrovascular diseases, severe cardiovascular diseases, metabolic diseases/disorders, alcohol addiction, malignant hyperthermia, or abnormal liver or renal function. This study adheres to the Strengthening the Reporting of Observational studies in Epidemiology (STROBE) guidelines.

### Management of general anesthesia

No preoperative medication was given. SPO_2_ (Pulse oxygen saturation), BP (Blood pressure), ECG (Electrocardiograph) and BIS (Bispectral index) were routinely monitored in all the patients. After pre-oxygen (8 L/min oxygen for approximately 3 min), the anesthetic circuit was primed with 8% sevoflurane. Patients inhaled 8% sevoflurane for anesthesia induction and tracheal intubation was facilitated with sufentanil (0.2–0.4 μg·kg^− 1^) and cis-atracurium (0.2–0.3 mg·kg^− 1^). Then, the lungs were ventilated with 50% oxygen balanced with air and end-tidal partial pressure of carbon dioxide (P_ET_CO_2_) was maintained between 35 and 45 mmHg. After endotracheal intubation, end-tidal sevoflurane concentration (ET_sevo_) was adjusted by turning volatile tank concentration up or down (0.5–1%) to maintain BIS value at 40–60 while continuous infusion of sufentanil with a rate of 0.1–0.2 μg·kg^− 1^·h^− 1^, adjusted according to vital signs. Patients received bolus of cis-atracurium and additional sufentanil according to clinical requirements during anesthesia maintenance. ET_sevo_ was automatically recorded using an anesthetic gas monitor (M1026B; Philips Medizin Systeme, Boblingen, Germany). Sevoflurane was discontinued approximately 10–20 min before the end of surgery. All patients were visited in the post-anesthesia care unit on postoperative day 1 and day 3 to assess intraoperative recall.

### Trial grouping

To investigate dose-dependent effect of sevoflurane on systemic metabolome, the patients were divided into high ET_sevo_ and low ET_sevo_ groups as following described. The patients in the high ET_sevo_ group might receive more sevoflurane than that of the patients in the low ET_sevo_ group during the surgery while their BIS values were similar. In total, 500 patients were enrolled. The mean value of ET_sevo_ from 20 min after endotracheal intubation to 2 h after the beginning of surgery was calculated for each patient, from which population ET_sevo_ was yielded. Low ET_sevo_ was denoted as ET_sevo_ value 1-time SD lower than population mean ET_sevo_. High ET_sevo_ was denoted as ET_sevo_ value 1-time SD higher than population mean ET_sevo_. For the low and high ET_sevo_ groups, we randomly paired 59 patients based on age, BMI (Body mass index), gender, surgical procedure (laparotomy or laparoscopy), BIS, vital signs and P_ET_CO_2_. After pairing, we divided the patients into the low ET_sevo_ group (group L) and high ET_sevo_ group (group H).

Peripheral venous blood was collected from all the patients before the induction of general anesthesia (before) and 2 h after the beginning of surgery (after). The blood samples were then centrifuged for 10 min at 3000 rpm at 4 °C, and the supernatant plasma was collected and frozen at − 80 °C. After identifying the patients in group L and H, their serum samples were retrieved and further divided into four groups based on ET_sevo_ grouping and sampling time as: L-before, L-after, H-before and H-after groups.

### Data processing and multivariate statistical analysis

In total, 100 μL of plasma and 300 μL of methanol were mixed. The mixture sonicated for 10 min and incubated for 1 h at − 20 °C. Then, the samples were centrifuged at 12,000 rpm for 15 min. The resulting supernatants were transferred to LC-MS vials and stored at − 80 °C. Quality control (QC) samples were prepared by mixing an equal aliquot of the supernatants from all of the samples. LC-MS/MS analysis was performed using an UHPLC system (1290, Agilent Technologies) with a UPLC HSS T3 column (2.1 mm × 100 mm, 1.8 μm) coupled to Q Exactive (Orbitrap MS, Thermo). Mobile phase A was 0.1% formic acid in water for the positive ion mode and 5 mmol·L^− 1^ ammonium acetate in water for the negative ion mode, and mobile phase B was acetonitrile. The QE mass spectrometer was used to acquire MS/MS spectra on an information-dependent basis during the LC/MS experiment. In this mode, the acquisition software (Xcalibur 4.0.27, Thermo) continuously evaluated the full-scan survey MS data. MS raw data from the UHPLC system were converted to the mzML format using ProteoWizard. Then, the data were filtered using the followed criterion: less than 50% all sample numbers in a group contained a metabolite (QC samples were also taken as a group). Next, missing values were replaced by half of the minimum value in the data set by default [14]. OSI-SMMS (Version 1.0, Dalian Chem Data Solution Information Technology Co. Ltd.) was used for the self-built database after XCMS (Version 3.2) data processing. The repeated metabolites from the positive and negative ion modes were merged.

Orthogonal projection to latent structures-discriminant analysis (OPLS-DA) was applied for between-group comparisons using R package models. The OPLS-DA model was further validated by cross-validation and permutation test. The predicted parameters of OPLS-DA model included R^2^X, R^2^Y and predictive ability (Q^2^) values. R^2^X and R^2^Y represent the interpretation rate of the model to X and Y matrices. Q^2^ values represented the most recognized diagnostic statistical parameter to validate the OPLS-DA model in metabolomics. Acceptable predictive model is considered for Q^2^ value greater than 0.5 [15].

### Differential metabolites and pathway analysis

The variable importance in projection (VIP) score of the OPLS-DA model was applied to rank the metabolites that best distinguished between comparisons. A *t*-test was also used for univariate analysis to screen differential metabolites. Metabolites with *P* < 0.05 and VIP ≥ 1 were considered differential metabolites between comparisons [16]. Metabolites were mapped to KEGG [17] metabolic pathways for pathway and enrichment analyses. *P* < 0.05 was statistical significance. Pathways meeting this condition were defined as significantly enriched pathways for differential metabolites.

### Statistical analysis

Statistical analyses were performed using SPSS (Version 22.0, IBM Corp., Armonk, NY, USA). Normally distributed data were presented as the mean ± SD, whereas non-normally distributed data were presented as the median and interquartile range. Analysis of variance was used for continuous variables. Categorical data were presented as numbers and compared using the Chi-squared test. Groups were compared using Student’s *t*-test (normally distributed data) or the Mann-Whitney U test (skewed data). To identify correlations between metabolite levels and the amount of sevoflurane exposure, stepwise multivariate linear regression was used, and Pearson’s correlation analysis was applied using MetaboAnalyst 4.0.

## Results

### Characteristics of patients

In total, 500 patients were enrolled, and no patient reported intraoperative recall. The population mean ET_sevo_ of the 500 patients was 1.60% ± 0.34%. Seventy-two patients were low ET_sevo_ (ET_sevo_ < 1.26%) (group L), and 102 patients were high ET_sevo_ (ET_sevo_ > 1.94%) (group H). According to the principle of pairing, we paired 55 patients from the group L and 59 patients from the group H. The mean ET_sevo_ of the group L was 1.06% ± 0.13%, which was significantly lower than that of group H (2.17% ± 0.16%, *P* < 0.001). All other primary characteristics of the patients were similar between the two groups (supplementary Table 1). The flow chart of the trial scheme is shown in supplementary Fig. 1.

### Multivariate statistical analysis

**Fig. 1:**
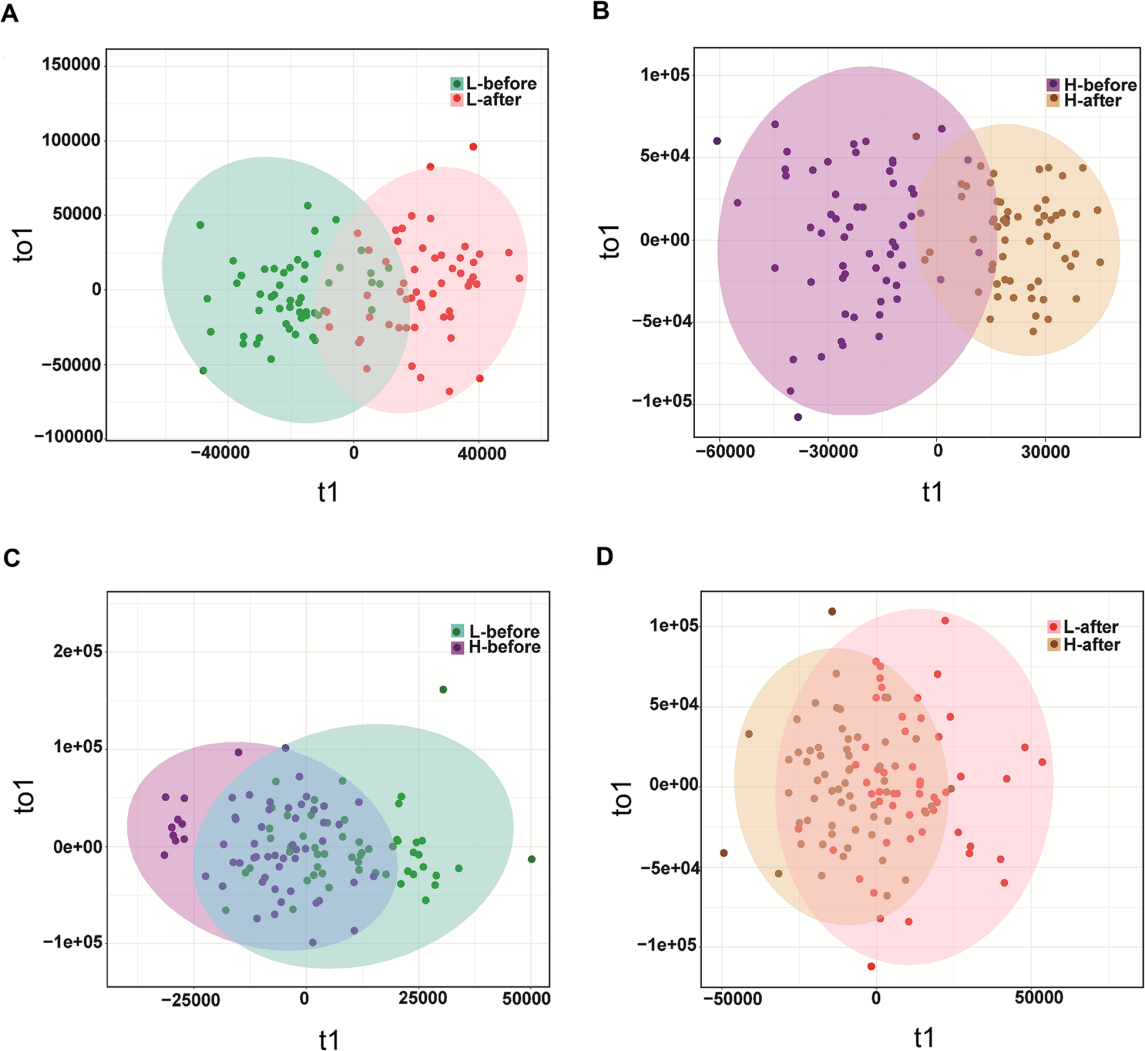
OPLS-DA score plots between the L-before and L-after groups and between the H-before and H-after groups (**a**, **b**). OPLS-DA score plots between the L-before and L-after groups and between the H-before and H-after groups (**c**, **d**)

Two OPLS-DA models were built using a dataset including the four group samples, with validation parameters as follows: Q^2^ = 0.592 (L-before vs. L-after, supplementary Table 2); Q^2^ = 0.667 (H-before vs. H-after, supplementary Table 2). The score plots revealed that each class was well separated, suggesting that the OPLS-DA model successfully discriminated samples according to their underlying metabolic profiles (Fig. 1a, b). However, the model data did not predict distinction at the same time point between groups as L-before vs. H-before and L-after vs. H-after (Q^2^ < 0.5) (supplementary Table 2). The distribution of sample dots between the two groups for L-before vs. H-before and L-after vs. H-after overlapped well, suggesting the levels of most metabolites were similar at the same time point between the two groups (Fig. 1c, d).

### Identification of potential regulated metabolites

For all selected patients, 2356 metabolites were significantly changed between before the induction of anesthesia and 2 h after the beginning of surgery, while 295 metabolites were detected after filters. In group L, 1558 metabolites were significantly changed between before the induction of anesthesia and 2 h after the beginning of surgery, while 93 metabolites were detected after filters. In group H, 1553 metabolites were significantly changed between before the induction of anesthesia and 2 h after the beginning of surgery and 91 metabolites were detected after filters. Compared to group H, 1562 metabolites were significantly changed before the induction of anesthesia in group L, but only 25 metabolites were identified after filters. Compared to group H, 1562 metabolites were significantly changed at 2 h after the beginning of surgery in group L, but only 38 metabolites were identified after filters. OPLS-DA was used to screen differential metabolites. Metabolites with *P* < 0.05 and VIP ≥ 1 were considered differential metabolites between comparisons [16]. For all selected patients, the levels of seventeen metabolites were significantly increased after anesthesia/abdominal surgery, while thirteen metabolites decreased (Table 1). According to the changed metabolites after anesthesia/abdominal surgery, sevoflurane anesthesia and abdominal surgery disturbed the tricarboxylic acid (TCA) cycle as identified by increased citrate and cis-aconitate levels, and impacted glycometabolism as identified by increased sucrose and D-glucose levels in all selected patients. Sevoflurane anesthesia and abdominal surgery also impacted the levels of other metabolites such as phenylethylamine, L-carnitine and nicotine.

**Table 1:** Up- and down-regulated metabolites between before anesthesia and 2 h after starting surgery in all patients

Metabolites	Fold change	***P***-value	q-value	VIP
N-Acetyl-D-glucosamine 6-phosphate	6.581917	2.67E-14	4.98E-13	2.902062
AMP	4.707389	2.42E-13	3.68E-12	1.145699
Ephedrine	1.888023	0.000189	0.000568	1.898164
PC (15:0/20:0)	1.775193	0.001708	0.004864	1.232962
Sucrose	1.712725	3.36E-05	0.000114	1.409021
4-Ketocyclophosphamide	1.494188	0.007404	0.015887	2.021637
Acyclovir	1.402124	4.21E-07	1.92E-06	1.021479
Se-Methyl-L-selenocysteine	0.826514	0.000576	0.001884	2.999008
D-erythro-1-(Imidazol-4-yl) glycerol-3-phosphate	0.73012	1.66E-10	1.92E-09	1.236423
Citrate	0.381647	8.53E-07	4.95E-06	4.650407
4,6-Dichloro-3-methylcatechol	0.361359	4.00E-06	2.06E-05	1.123057
L-ribulose	0.333307	4.88E-12	5.66E-11	1.20026
cis-Aconitic acid	0.316411	1.42E-05	6.69E-05	1.128694
D-glucose	0.303546	5.18E-12	5.95E-11	6.04479
L-glutamine	0.225095	2.67E-10	2.44E-09	1.856811
L-selenocysteine	0.210814	1.33E-08	7.61E-08	2.994583
Pyroglutamic acid	0.150226	7.92E-05	0.063248	1.814755
Se-Methylselenomethionine	−5.14371	1.02E-18	3.19E-07	1.97583
Phenylethylamine	−1.99326	4.77E-26	5.97E-24	2.200773
Glycochenodeoxycholic acid	−1.42167	0.000184	0.000555	1.278177
5-Acetylamino-6-formylamino-3-methyluracil	−0.84274	4.91E-14	7.87E-13	1.313026
Cholesterol sulfate	−0.59075	9.72E-08	6.45E-07	1.349612
Bilirubin	−0.37302	0.000103	0.000417	1.323645
LysoPC (18:2(9Z,12Z))	−0.36135	8.93E-11	7.23E-10	12.17143
dTDP-3-methyl-4-oxo-2,6-dideoxy-L-allose	−0.31602	2.38E-07	1.13E-06	3.282894
Nicotine	−0.28852	1.49E-10	1.17E-09	3.286386
L-carnitine	−0.28411	1.25E-10	9.95E-10	11.95738
2-Oxoglutaramate	−0.25893	4.79E-05	0.000167	1.722153
Sphinganine	−0.23381	3.38E-10	2.54E-09	1.935018
4′-O-Demethylrebeccamyc	−0.16481	0.002641	0.007141	2.88872

*VIP* Variable importance in the projection. Positive values in fold change indicate increases while negative values indicate decreases. q-value: False discovery rate adjusted *p*-values

**Fig. 2:**
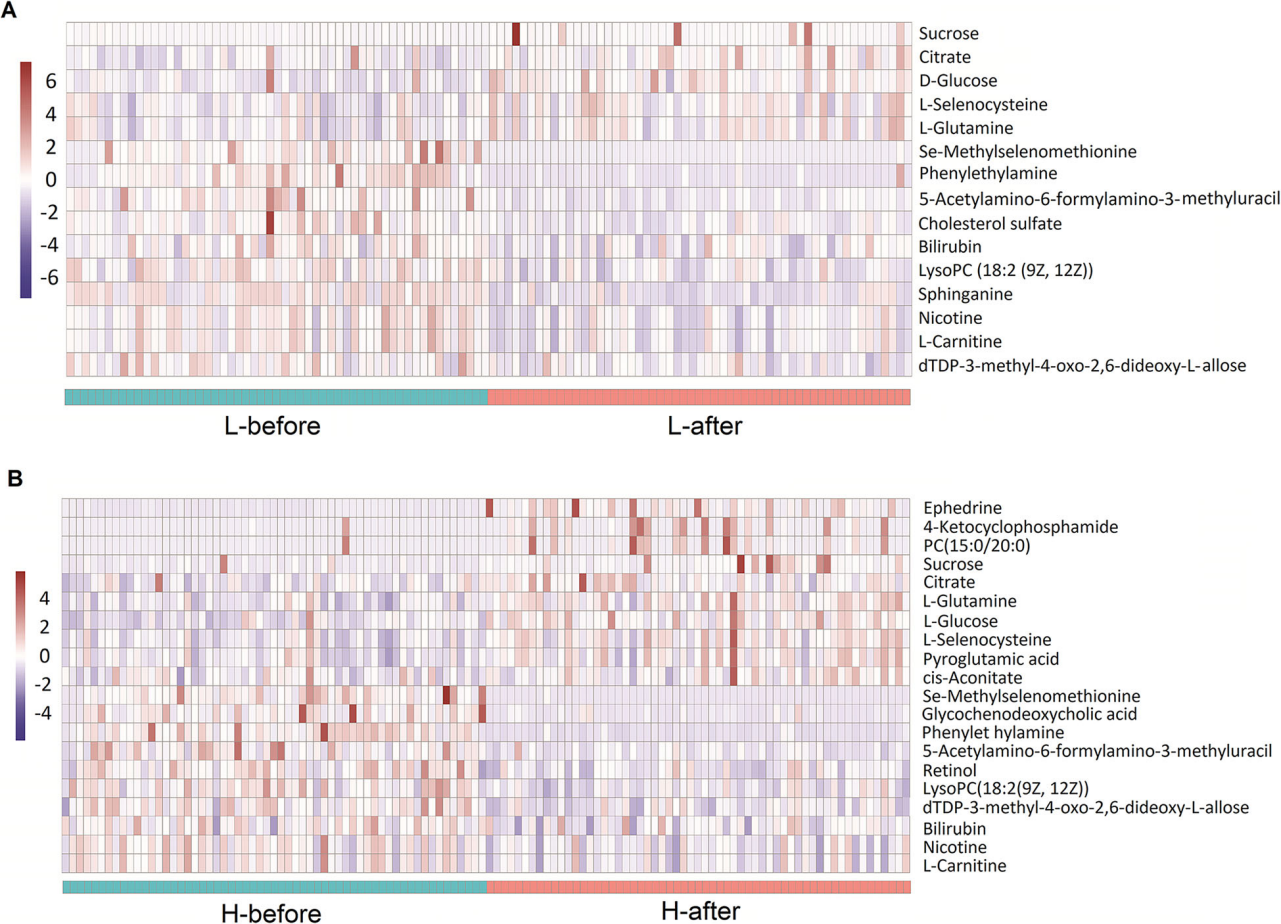
Up- and down-regulated metabolites between the L-before and L-after groups and between the H-before and H-after groups. **a** Heatmap of 15 metabolites with significantly different levels (5 increased and 10 decreased) between the L-before and L-after groups for individual patients. **b** Heatmap of 20 metabolites with significantly different levels (10 increased and 10 decreased) between the H-before and H-after groups for individual patients

The significantly changed metabolites within the subgroups (group L or group H) before and after anesthesia/abdominal surgery were also analysed and presented as heatmaps in Fig. 2. In the group L, the levels of five metabolites were significantly increased after anesthesia/abdominal surgery, whereas ten metabolites decreased (supplementary Table 3). In the group H, the levels of ten metabolites were significantly increased after anesthesia/abdominal surgery, while thirteen metabolites decreased (supplementary Table 4).

The significantly changed metabolites at the same time point between group L and H were presented as heatmaps in supplementary Fig. 2. Before anesthesia, compared with group L, only one metabolite was higher and two metabolites were lower in group H (supplementary Table 5). After anesthesia/abdominal surgery, the levels of seven metabolites were higher in group H than those in group L, whereas three metabolites were lower in group H (supplementary Table 6).

**Fig. 3:**
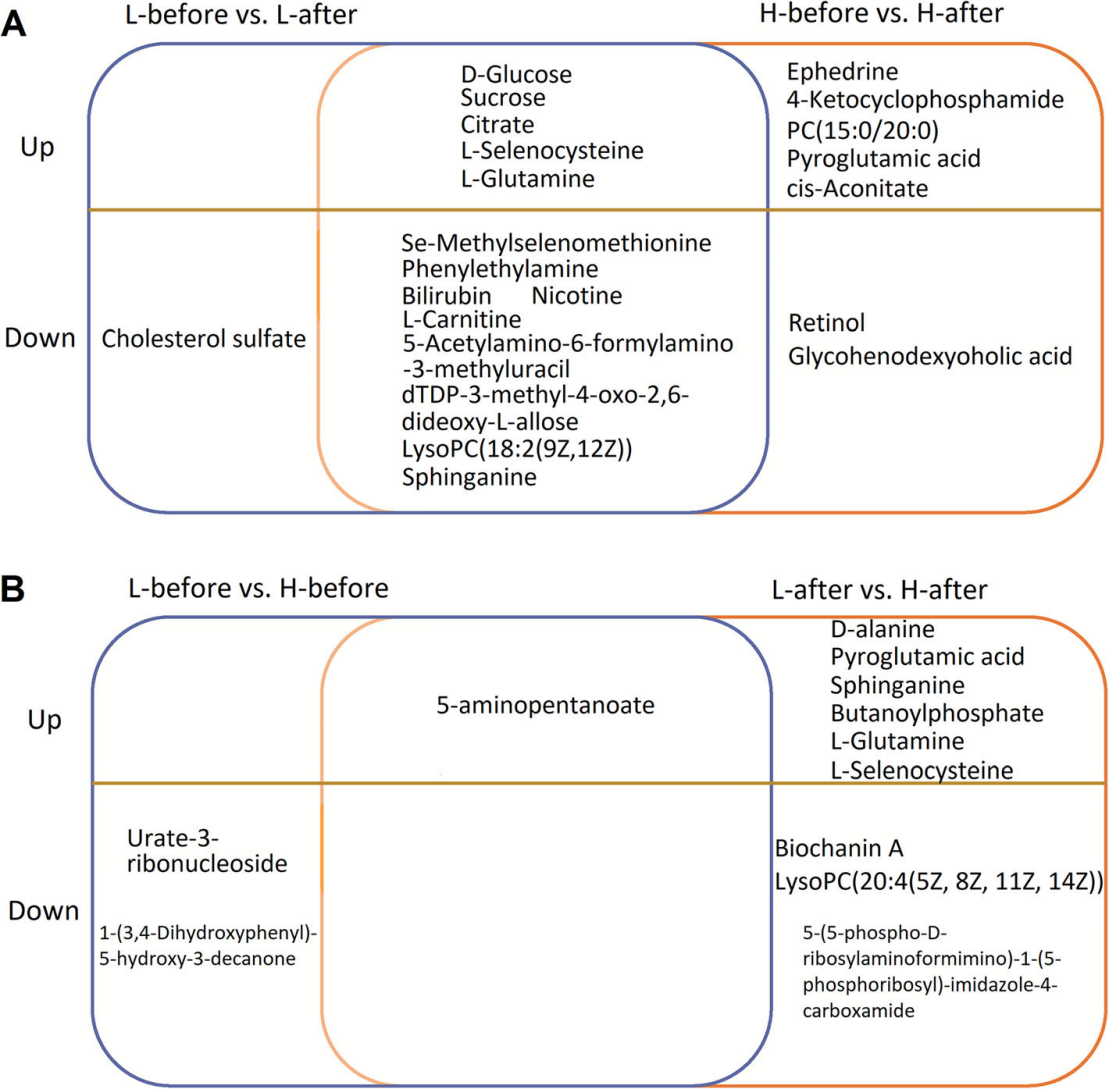
Up- and down-regulated metabolites among the L-before, L-after, H-before and H-after groups. **a** Up- and down-regulated metabolites between the L-before and L-after groups and between the H-before and H-after groups. **b** Up- and down-regulated metabolites between the L-before and H-before groups and between the L-after and H-after groups

Theoretically, changed metabolites of all selected patients (Table 1) or common changed metabolites of group H and group L after anesthesia/abdominal surgery mainly represent the metabolic pathways impacted by anesthesia/abdominal surgery (Fig. 3a). While the differentially changed metabolites after anesthesia/abdominal surgery between group H and group L may represent the metabolic pathways relevant to the amount of sevoflurane exposure or sevoflurane sensitivity (Fig. 3b). Our results showed that the levels of 5-aminopentanoate, L-glutamine, pyroglutamic acid, L-selenocysteine and sphinganine in group L after anesthesia/abdominal surgery were higher than those in group H.

### Biological pathway analysis

Pathway analysis was applied to investigate biological functions of the altered metabolites. Multiple metabolic pathways were impacted after anesthesia/abdominal surgery in both group L (supplementary Fig. 3A) and group H (supplementary Fig. 3B), mainly including carbohydrate digestion and absorption, ABC transporters, mineral absorption, glutamate metabolism. Interestingly, some metabolic pathways were only impacted in H group (supplementary Fig. 3B) such as glutamatergic synapse and GABAergic synapse, which might account for higher requirement of sevoflurane for these patients or higher amount of sevoflurane exposure. The involvement of the altered metabolites in KEGG pathways between L and H groups at the same time (before or after anesthesia/abdominal surgery) was shown in supplementary Fig. 4*.*

### Changes of four metabolites after anesthesia/abdominal surgery are relevant to the amount of sevoflurane exposure

**Fig. 4:**
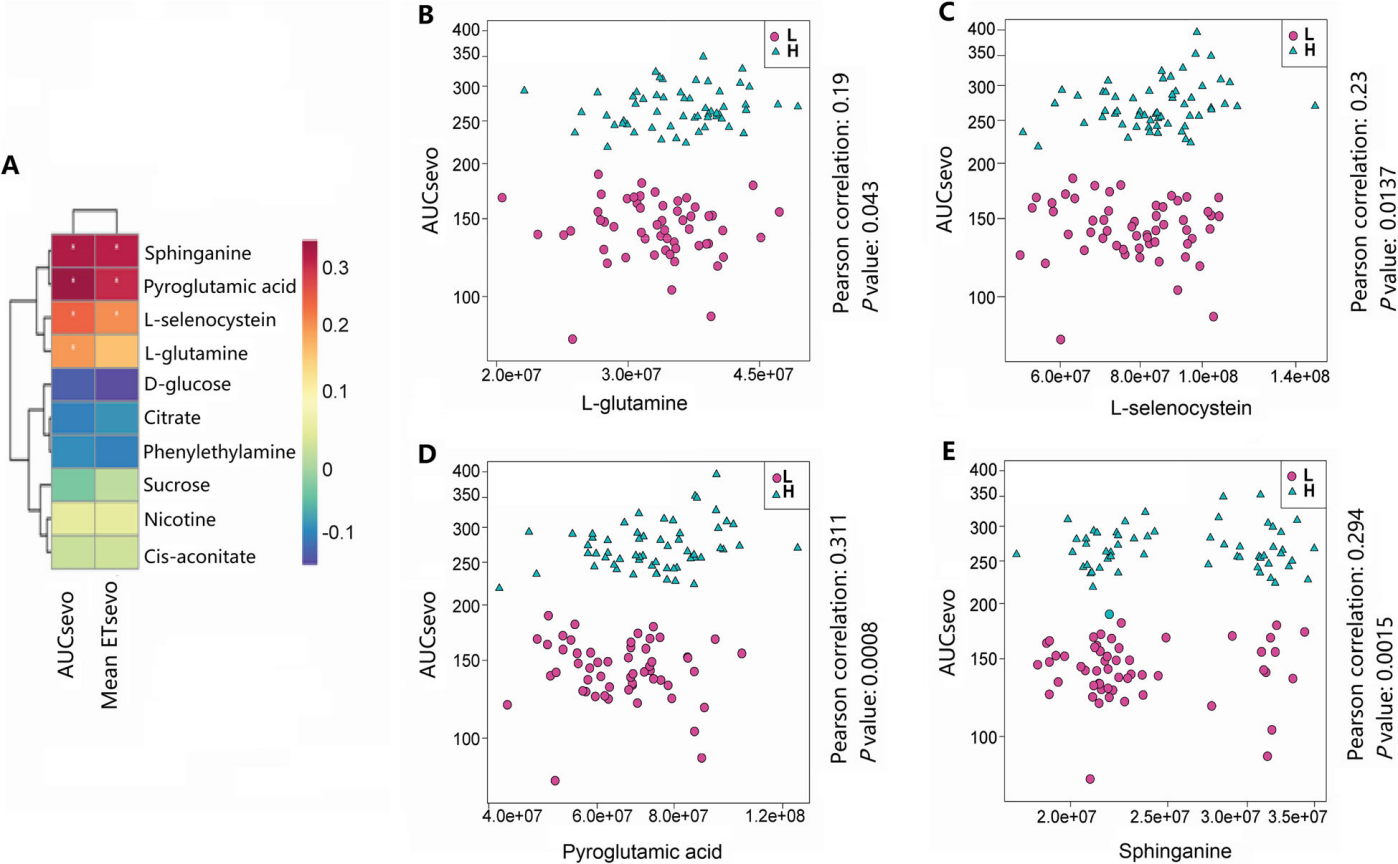
Correlation analysis between metabolites and AUC-ET_sevo_. **a** Correlation analysis between 10 metabolites and AUC-ET_sevo_. Four metabolites, including L-glutamine (**b**), L-selenocysteine (**c**), pyroglutamic acid (**d**) and sphinganine (**e**) were correlated with AUC-ET_sevo_

Glutamate metabolism might be critical for sevoflurane anesthesia and sensitivity. L-glutamine, L-selenocysteine, pyroglutamic acid involve in glutamate metabolism. Sevoflurane anesthesia and abdominal surgery increased L-glutamine, pyroglutamic acid, L-glutamine, pyroglutamic acid and 5-aminopentanoate levels in the group H compared with that those in the group L. Sevoflurane anesthesia and abdominal surgery decreased sphinganine level, and its level was higher in the H group after anesthesia. Correlation analysis identified four metabolites that may be relevant to the sevoflurane requirement (Area under curve of ET_sevo_, Fig. 4a): L-glutamine (Fig. 4b, P = 0.0435), L-selenocysteine (Fig. 4c, P = 0.0137), pyroglutamic acid (Fig. 4d, P = 0.0008) and sphinganine (Fig. 4e, P = 0.0015). These higher increased metabolites in the group H after anesthesia/abdominal surgery may result from higher amount of sevoflurane exposure. In turn, such differential metabolic status might determine sevoflurane requirement.

## Discussion

The present study indicated that sevoflurane anesthesia and abdominal surgery can significantly impact major metabolic pathways in human. Sevoflurane and abdominal surgery increased citrate, cis-aconitate, sucrose and D-glucose levels in patients. Glutamate metabolism was also impacted by sevoflurane and abdominal surgery. In the patients with high ETsevo, levels of L-glutamine, pyroglutamic acid, sphinganine and L-selenocysteine after sevoflurane anesthesia and abdominal surgery were significantly higher than those of the patients with low ETsevo, suggesting that these metabolic changes might be relevant to the amount of sevoflurane exposure.

Sevoflurane anesthesia has been known to impact the systemic metabolism in animals. For example, sevoflurane anesthesia increases the blood concentration of glucose and decreases blood concentration of lactate in rodents [18]. Lipid metabolism is also disturbed after sevoflurane anesthesia in monkeys, for example, lipid species including polyunsaturated fatty acids are depleted after sevoflurane anesthesia [19]. However, how sevoflurane anesthesia and surgery impact systemic metabolomics in clinical patients is largely unknown.

In this study, we compared alteration of systemic metabonomics after sevoflurane anesthesia and abdominal surgery in human subjects. Our results indicate that sevoflurane anesthesia and abdominal surgery can impact principal metabolic pathways, including the TCA cycle, glycometabolism and glutamate metabolism. The TCA cycle is the final common oxidative pathway connecting all individual metabolic pathways [20]. In this cycle, citrate is converted to isocitrate via cis-aconitate by aconitase [20], and citrate can be exported from the mitochondria through citrate carriers [20]. Citrate links many important cellular processes, bridging carbohydrate and fatty acid metabolism and protein modification. The results here indicated that citrate and cis-aconitate were accumulated after sevoflurane anesthesia, suggesting that the TCA cycle might be impeded during general anesthesia.

Previous studies of animals indicate that sevoflurane may disrupt glucose metabolism [2], insulin secretion [21], insulin resistance and glucose uptake [2, 6]. Elevated glucose level is associated with increased infection rates after general surgeries [22]. The expression of several pro-inflammatory cytokines is regulated by glucose levels, including tumour necrosis factor TNF-α [23] and interleukin IL-1β [10]. The impairment of neutrophil degranulation induced by hyperglycaemia is also relevant to infection after surgery [10]. Our results indicated that sevoflurane anesthesia and abdominal surgery increased sucrose and D-glucose levels in both groups, which might increase the infection risks of postoperative patients.

Alteration of metabonomics after anesthesia and surgery may also contribute to postoperative cognitive dysfunction (POCD) [24]. Previous study indicates that injection of nicotine after anesthesia induction can markedly prevent POCD in rats [25]. L-carnitine can exert neuroprotective effects via attenuating inflammation and oxidative damage [26]. Low *phenylethylamine* level in children is relevant to attention deficit-hyperactivity disorder [27]. The concentration of *phenylethylamine* has been proposed as a useful biochemical marker in psychiatric and behavioural research [28]. Our results showed that the content of nicotine, L-carnitine and phenylethylamine in plasma decreased after anesthesia/abdominal surgery compared with those before anesthesia in both groups, which may impact postoperative cognitive functions of patients.

Interestingly, correlation analysis identified four metabolites potentially relevant to the amount of sevoflurane exposure: L-glutamine, pyroglutamic acid, sphinganine and L-selenocysteine. L-glutamine is involved in glutamate metabolism and glutamate is an essential metabolic precursor in glucose biosynthesis, protein synthesis and glutathione homeostasis [29]. Glutamate is the most abundant excitatory neurotransmitter in the brain and plays a fundamental role in learning and memory [30]. Glutamate released by pre-synaptic neurons is rapidly converted to glutamine in astrocytes and glutamine released from astrocytes can also be converted back to glutamate. The glutamate/glutamine cycle is essential for normal function of synaptic transmission [30]. Depression of excitatory synaptic transmission is considered the principal mechanism of general anesthesia [31]. The level of brain glutamine and glutamate were also altered in dogs when anesthetized with sevoflurane and isoflurane [3]. Volatile anesthetics may increase glutamate uptake by astrocytes, thereby affecting excitatory synaptic transmission in the central nervous system [32]. Our results indicated that the level of L-glutamine was higher in the group H after anesthesia/abdominal surgery than that in the group L, which might be related to the differential functions in excitatory synaptic transmission and excitatory neurotransmitter release during sevoflurane anesthesia. This may also result from the different requirement of sevoflurane between the group L and group H.

Pyroglutamic acid, derived from L-glutamic acid in the γ-glutamyl cycle, is one of the essential components for amino acid transport [33]. Pyroglutamic acid induces anti-diabetic properties in vivo and inhibits energy production of the cerebral cortex of rats [34]. Selenocysteine is involved in glutamate metabolism, DNA damage and can inhibit cell growth and mitochondrial function by triggering reactive oxygen species [35]. The inhibition of mitochondrial function is one of the most important mechanisms by which volatile anesthetics exert their anesthetic effect [36]. Our results indicated that the content of pyroglutamic acid and L-selenocysteine in the group H after anesthesia/abdominal surgery were higher than those in the group L, which may be related to the differential glutamate metabolism during sevoflurane anesthesia.

Sphinganine, an important structural element of cell membranes [37], plays a key role in the biosynthesis and metabolism of sphingolipids [38]. Altered sphingolipid metabolism is potentially related to the inflammatory response and increased ceramide levels [38]. Studies has shown that the reduction of sphinganine in cortical neurons can impact mitochondrial respiratory function [37]. However, the effects of anesthesia/surgery on sphinganine level has not been reported. Our results indicate that the level of sphinganine in the group H after anesthesia and abdominal surgery were higher than that in the group L.

The present study has several limitations. Firstly, we cannot separately investigate the effect of sevoflurane or abdominal surgery on metabonomics in patients. Therefore, the systemic effects on metabonomics here are the overall effects. Secondly, although the central nervous system is the main target for general anesthetics, we cannot measure the change of metabonomics in the brain.

## Conclusions

In summary, the present study indicated that sevoflurane anesthesia and abdominal surgery can impact principal metabolic pathways in clinical patients. The plasma concentrations of L-glutamine, pyroglutamic acid, sphinganine and L-selenocysteine might be relevant to the amount of sevoflurane exposure. This study may provide a resource data for future studies about metabolism relevant to general anaesthesia and surgeries.

## Supplementary Information

**Additional file 1: Supplementary Table 1.** Characteristics of the study patients. **Supplementary Table 2.** Permutation test results of the OPLS-DA models. **Supplementary Table 3.** Top up- and down-regulated metabolites in the group L before and after sevoflurane anesthesia/surgery. **Supplementary Table 4.** Top up- and down-regulated metabolites in the group H before and after sevoflurane anesthesia/surgery. **Supplementary Table 5.** Up- and down-regulated metabolites between L-before group and H-before group. **Supplementary Table 6.** Up- and down-regulated metabolites between L-after group and H-after group after sevoflurane anesthesia and surgery. **Supplementary Figure 1.** Flow diagram of the clinical trial. **Supplementary Figure 2.** Up- and down-regulated metabolites between the L vs. H groups. **(**A) Heatmap of 12 metabolites with significantly different levels between the L-before and H-before groups. (B) Heatmap of 15 metabolites with significantly different levels between the L-after and H-after groups. **Supplementary Figure 3.** Bubble plot of pathway analysis. (A) The pathways that differed between the L-before and L-after groups. (B) The pathways that differed between the H-before and H-after groups. Each dot represents a related metabolic pathway. The colour and size of each dot denote the −ln(p) value and pathway impact value, respectively. **Supplementary Figure 4.** Bubble plot of pathway analysis. (A) The pathways that significantly differed between the L-before and H-before groups. (B) The pathways that significantly differed between the L-after and H-after groups. Each dot represents a related metabolic pathway. The colour and size of each dot denote the−ln(p) value and pathway impact value, respectively.

## Data Availability

The datasets used and/or analysed during the current study are available from the corresponding author on reasonable request.

## Abbreviations

ET_sevo_: End-tidal sevoflurane concentration
BIS: Bispectral index
SPO_2_: Pulse oxygen saturation
BP: Blood pressure
ECG: Electrocardiograph
BIS: Bispectral index
P_ET_CO_2_: End-tidal partial pressure of carbon dioxide
BMI: Body mass index
QC: Quality control
OPLS-DA: Orthogonal projection to latent structures-discriminant analysis
VIP: Variable importance in projection
TCA: Tricarboxylic acid
POCD: Postoperative cognitive dysfunction
AUC: Area under curve
