# Supplementary material for: Effects of sevoflurane anesthesia and abdominal surgery on the systemic metabolome: a prospective observational study
Source: BMC Anesthesiol. 2021 Mar 17;21:80. doi: 10.1186/s12871-021-01301-0 (PMC7968205; doi:10.1186/s12871-021-01301-0)
Supplement: Supplementary file 1 — Additional file 1: Supplementary Table 1. Characteristics of the study patients. Supplementary Table 2. Permutation test results of the OPLS-DA models. Supplementary Table 3. Top up- and down-regulated metabolites in the group L before and after sevoflurane anesthesia/surgery. Supplementary Table 4. Top up- and down-regulated metabolites in the group H before and after sevoflurane anesthesia/surgery. Supplementary Table 5. Up- and down-regulated metabolites between L-before group and H-before group. Supplementary Table 6. Up- and down-regulated metabolites between L-after group and H-after group after sevoflurane anesthesia and surgery. Supplementary Figure 1. Flow diagram of the clinical trial. Supplementary Figure 2. Up- and down-regulated metabolites between the L vs. H groups. (A) Heatmap of 12 metabolites with significantly different levels between the L-before and H-before groups. (B) Heatmap of 15 metabolites with significantly different levels between the L-after and H-after groups. Supplementary Figure 3. Bubble plot of pathway analysis. (A) The pathways that differed between the L-before and L-after groups. (B) The pathways that differed between the H-before and H-after groups. Each dot represents a related metabolic pathway. The colour and size of each dot denote the −ln(p) value and pathway impact value, respectively. Supplementary Figure 4. Bubble plot of pathway analysis. (A) The pathways that significantly differed between the L-before and H-before groups. (B) The pathways that significantly differed between the L-after and H-after groups. Each dot represents a related metabolic pathway. The colour and size of each dot denote the−ln(p) value and pathway impact value, respectively. [file 12871_2021_1301_MOESM1_ESM.docx]

**Title:** Effects of sevoflurane anesthesia and abdominal surgery on the systemic metabolome: a prospective observational study

**Running title:** Metabolome after sevoflurane and abdominal surgery

Yiyong Wei ^1, 2 #^, Donghang Zhang ^1, 2 #^, Jin Liu ^1, 2 *^, Mengchan Ou ^1, 2^, Peng Liang ^2^, Yunxia Zuo ^2 *^, Cheng Zhou ^1^

^1^ Laboratory of Anesthesia & Critical Care Medicine, Translational Neuroscience Center, West China Hospital of Sichuan University, Chengdu 610041, China;

^2^ Department of Anesthesiology, West China Hospital of Sichuan University, Chengdu 610041, China;

^#^ Yiyong Wei and Donghang Zhang contributed equally to this study.

* **Corresponding authors**

Dr. Jin Liu,

Laboratory of Anesthesia & Critical Care Medicine, Translational Neuroscience Center, West China Hospital of Sichuan University, 37# Guoxue Xiang, Chengdu 610041, Sichuan, China;

E-mail: [scujinliu@gmail.com](mailto:scujinliu@gmail.com)

Dr. Yunxia Zuo,

Department of Anesthesiology, West China Hospital of Sichuan University, 37# Guoxue Xiang, Chengdu 610041, Sichuan, China;

E-mail: [zuoyunxia@scu.edu.cn](mailto:zuoyunxia@scu.edu.cn)

**Supplementary Tables:**

Supplementary Table 1: Characteristics of the study patients.

| **Characteristics** | **L group (n = 55)** | **H group (n = 59)** | **P-value** |
| --- | --- | --- | --- |
| Age (Years) | 52.49 ± 8.759 | 53.67 ± 11.06 | 0.270 |
| Gender (M/F) | 34/21 | 40/19 | 0.634 |
| BMI (kg m^-2^) | 22.38 ± 3.09 | 23.05 ± 1.93 | 0.125 |
| ASA (Ⅰ～Ⅱ) | 2/53 | 1/58 | 0.518 |
| MAP (mmHg) | 87.43 ± 9.54 | 80.69 ± 2.62 | 0.171 |
| HR (Beats min^-1^) | 71.37 ± 9.18 | 74.17 ± 2.65 | 0.104 |
| P_ET_CO_2_ (mmHg) | 39.24 ± 4.62 | 37.47 ± 5.63 | 0.683 |
| Mean BIS  Area under BIS-time curve | 51.32 ± 3.19  6579.0±115.7 | 45.00 ± 3.25  6413.0±135.4 | 0.076  0.156 |
| Sufentanil (Induction) (μg) | 20.51 ± 4.34 | 20.00 ± 0.00 | 0.194 |
| Cis-atracurium (Induction) (mg) | 13.81 ± 2.86 | 14.67 ± 0.58 | 0.628 |
| Sufentanil (Until to surgery 2 hours) (μg) | 33.13 ± 8.44 | 35.00 ± 7.55 | 0.462 |
| Cis-atracurium (Until to surgery 2 hours) (mg) | 11.92 ± 4.20 | 10.67 ± 5.03 | 0.471 |
| Duration of anesthesia (min) | 184.20 ± 69.6 | 237.6 ± 31.8 | 0.175 |
| Mean ET_sevo_ (%)  Area under ET_sevo_-time curve (AUC-ET_sevo_) | 1.060 ± 0.129  136.8 ± 2.761 | 2.170 ± 0.161 ^*^  273.6 ± 4.84 ^*^ | 0.001  0.0001 |
| Tracheal extubation time (min) | 6 (3, 8) | 7 (4, 12) ^*^ | 0.010 |
| Stomach cancer  (Open/laparoscopic) | 15/2 | 17/2 | 0.906 |
| Colorectal cancer  (Open/laparoscopic) | 26/12 | 27/13 | 0.931 |

^*^ *P* < 0.05 vs. the L group by t-test. L group: The group of low mean ET_sevo_; H group: The group of high mean ET_sevo_; BMI: Body mass index; MAP: Mean arterial pressure; HR: Heart rate; P_ET_CO_2_: End-tidal partial pressure of carbon dioxide; BIS: Bispectral index; ET_sevo_: End-tidal concentration of sevoflurane. Data are mean ± SD or mean (minimum, maximum).

Supplementary Table 2: Permutation test results of the OPLS-DA models.

|  | **R^2^X** | **R^2^Y** | **Q^2^** |
| --- | --- | --- | --- |
| L-before/L-after | 0.277 | 0.688 | 0.592 |
| H-before/H-after | 0.313 | 0.732 | 0.667 |
| L-before/H-before | 0.334 | 0.378 | 0.058 |
| L-after/H-after | 0.267 | 0.359 | 0.067 |

R^2^X, the interpretability of the model for the categorical variable X;

R^2^Y, the interpretability of the model for the categorical variable Y；

Q^2^ values，predictability of the model.

Supplementary Table 3: Top up- and down-regulated metabolites in the group L before and after sevoflurane anesthesia/surgery.

| **Metabolites** | **Fold change** | **P-value** | **q-value** | **VIP** |
| --- | --- | --- | --- | --- |
| Sucrose  Citrate  D-Glucose  L-Selenocysteine  L-Glutamine  Se-Methylselenomethionine  Phenylethylamine  5-Acetylamino-6-formylamino-3-methyluracil  Cholesterol sulfate  Bilirubin  LysoPC (18:2(9Z,12Z))  Sphinganine  Nicotine  L-Carnitine  dTDP-3-methyl-4-oxo-2,6-dideoxy-L-allose | 1.82625  0.428713  0.330948  0.166729  0.145934  −4.80318  −1.91203  −0.95394  −0.75919  −0.44042  −0.36633  −0.35483  −0.32569  −0.32018  −0.29225 | 0.011185  0.000282  2.26E-06  0.001313  0.001933  3.43E-09  3.40E-12  7.60E-09  2.53E-05  0.003609  7.22E-06  2.67E-11  2.43E-06  2.14E-06  0.002263 | 0.031142  0.001263  1.65-E05  0.00485  0.00674  8.50E-08  3.32E-10  1.58E-07  0.000141  0.011697  4.59E-05  1.54E-09  1.75E-05  1.57E-05  0.007772 | 1.425573  3.508937  3.117985  1.131164  1.212773  1.295593  1.462387  1.280408  1.574171  1.546234  12.61681  1.874423  2.465473  8.976843  3.183852 |

VIP: Variable importance in the projection. Positive values in fold change indicate increases while negative values indicate decreases. q-value: False discovery rate adjusted p-values.

Supplementary Table 4: Top up- and down-regulated metabolites in the group H before and after sevoflurane anesthesia/surgery.

| **Metabolites** | **Fold change** | | **P-value** | **q-value** | **VIP** |
| --- | --- | --- | --- | --- | --- |
| Ephedrine  4-Ketocyclophosphamide  PC (15:0/20:0)  Sucrose  Citrate  L-Glutamine  D-Glucose  L-Selenocysteine  Pyroglutamic acid  cis-Aconitate  Se-Methylselenomethionine  Glycochenodeoxycholic acid  Phenylethylamine  5-Acetylamino-6-formylamino-3-methyluracil  Retinol  LysoPC (18:2(9Z,12Z))  dTDP-3-methyl-4-oxo-2,6-dideoxy-L-allose  Bilirubin  Nicotine  L-carnitine  2-oxoglutaramate  LysoPC (20:4(5Z,8Z,11Z,14Z))  Sphinganine | | 3.86349  3.675675  3.495701  1.625082  0.335318  0.309872  0.276554  0.249612  0.226588  0.143062  −5.60101  −2.2203  −2.0781  −0.7393  −0.44728  −0.35633  −0.33817  −0.31173  −0.25454  −0.25119  −0.23727  −0.18619  −0.12577 | 4.75E-08  4.63E-05  0.000263  0.000705  0.000936  1.61E-12  1.95E-07  1.54E-06  2.56E-05  0.024225  4.83E-11  1.46E-06  1.78E-15  1.37E-06  7.80E-06  2.16E-06  1.64E-05  0.011044  1.72E-05  1.62E-05  0.001639  0.002096  0.013749 | 7.13E-07  0.000255  0.001231  0.003000  0.003890  7.37E-11  2.29E-06  2.60E-06  1.38E-05  0.000154  1.68E-09  1.33E-05  1.63E-63  1.26E-05  5.67E-05  1.87E-05  0.000104  0.032232  0.000109  0.000103  0.006321  0.007795  0.377431 | 1.950325  2.060346  1.126578  1.170347  3.204581  1.736849  3.930996  2.05717  1.640917  1.472725  1.401231  1.538738  1.592256  1.104154  1.020765  12.56206  3.775363  1.183019  2.522879  9.234257  1.374603  1.028204  1.056136 |

VIP: Variable importance in the projection. Positive values in fold change indicate increases while negative values indicate decreases. q-value: False discovery rate adjusted p-values.

Supplementary Table 5: Up- and down-regulated metabolites between L-before group and H-before group.

| **Metabolites** | **Fold change** | **P-value** | **q-value** | **VIP** |
| --- | --- | --- | --- | --- |
| 5-aminopentanoate  Urate-3-ribonucleoside  1-(3,4-Dihydroxyphenyl)-5-hydroxy-3-decanone | 0.14432  −0.29431  −0.19227 | 0.046528  0.032742  0.048344 | 0.45140  0.37575  0.45391 | 12.62484  1.199299  1.278434 |

VIP: Variable importance in the projection. Positive values in fold change indicate increases while negative values indicate decreases. q-value: False discovery rate adjusted p-values.

Supplementary Table 6: Up- and down-regulated metabolites between L-after group and H-after group after sevoflurane anesthesia and surgery.

| **Metabolites** | **Fold change** | **P-value** | **q-value** | **VIP** |
| --- | --- | --- | --- | --- |
| D-alanine  5-aminopentanoate  Pyroglutamic acid  Sphinganine  Butanoylphosphate  L-Glutamine  L-Selenocysteine  5-(5-Phospho-D-ribosylaminoformimino)-1-  (5-phosphoribosyl)-imidazole-4-carboxamide  Biochanin A  LysoPC (20:4(5Z,8Z,11Z,14Z)) | 0.290996  0.249935  0.181161  0.176853  0.175064  0.129375  0.124462  −0.39995  −0.18304  −0.15575 | 1.11E-050.001161  0.001196  0.001013  0.001909  0.007362  0.017515  0.006409  0.021965  0.014745 | 0.007315  0.049031  0.049152  0.046527  0.056247  0.111648  0.192317  0.102147  0.215607  0.175216 | 1.992767  17.61254  3.079663  1.635978  1.054723  3.608327  2.976846  1.077645  2.554265  1.057573 |

VIP: Variable importance in the projection. Positive values in fold change indicate increases while negative values indicate decreases. q-value: False discovery rate adjusted p-values.

**Supplementary Figures:**


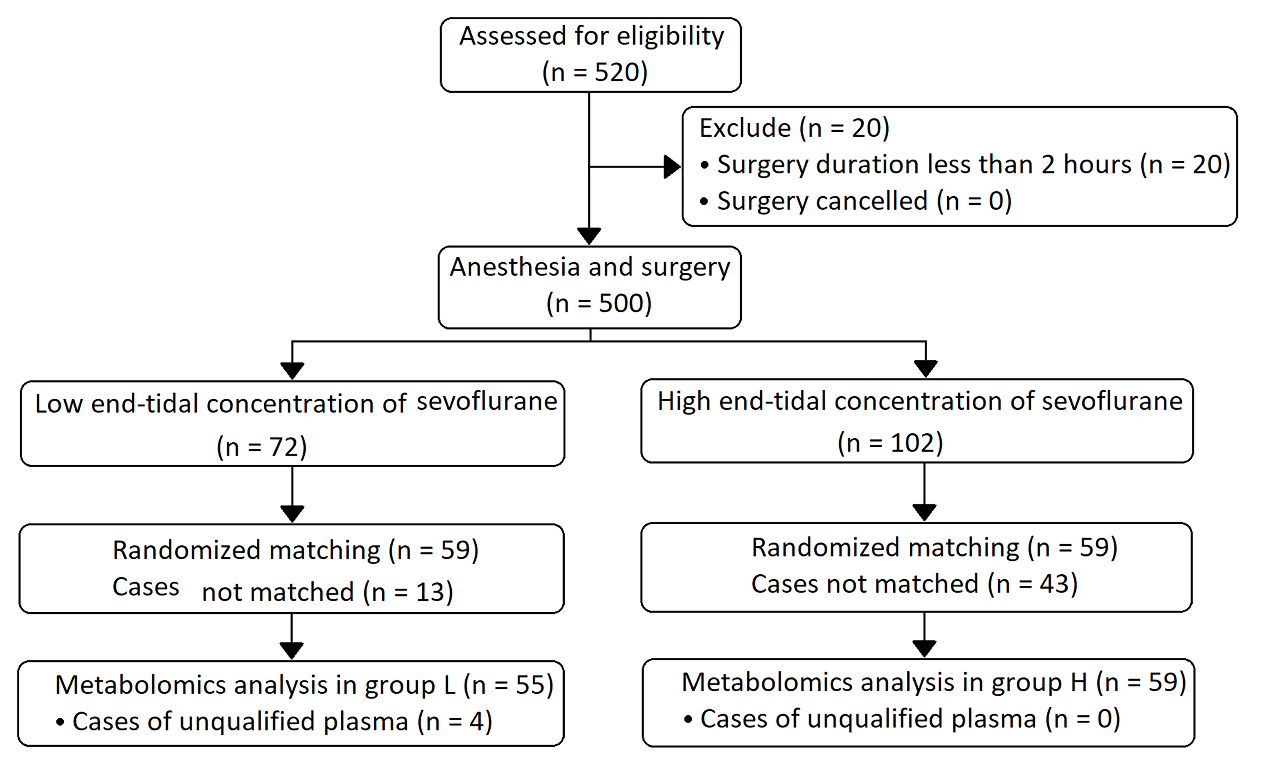


Supplementary Figure 1. Flow diagram of the clinical trial.


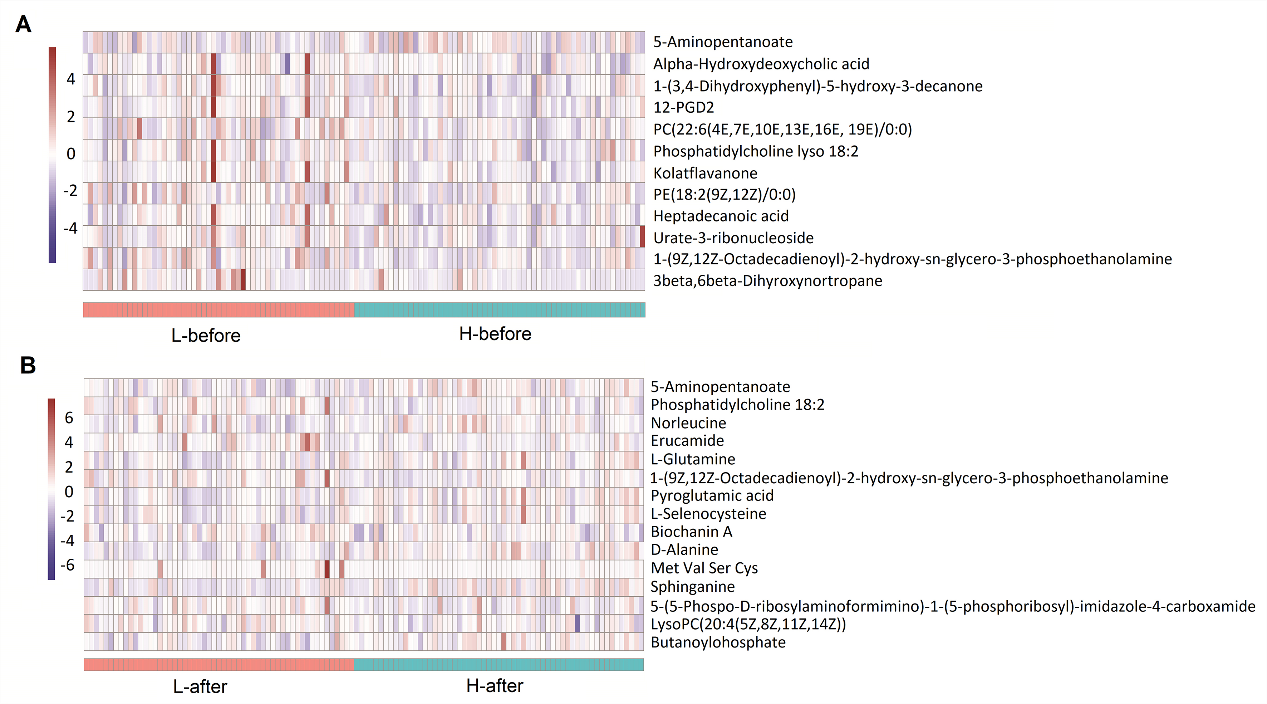


Supplementary Figure 2. Up- and down-regulated metabolites between the L vs. H groups. (A) Heatmap of 12 metabolites with significantly different levels between the L-before and H-before groups. (B) Heatmap of 15 metabolites with significantly different levels between the L-after and H-after groups.

**
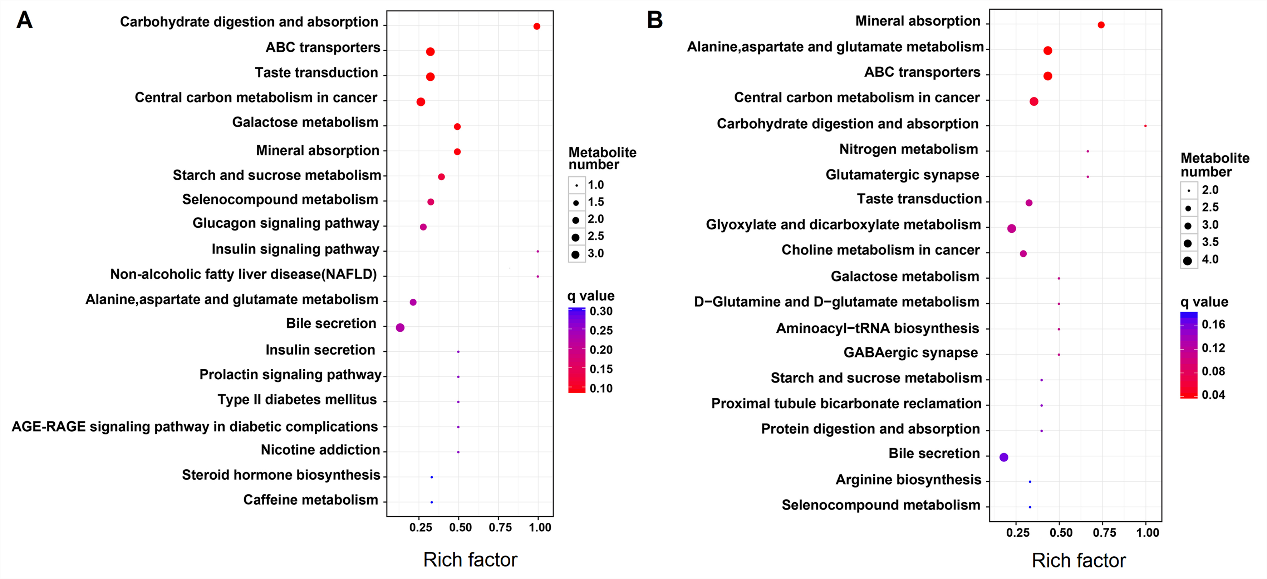
**

Supplementary Figure 3. Bubble plot of pathway analysis. (A) The pathways that differed between the L-before and L-after groups. (B) The pathways that differed between the H-before and H-after groups. Each dot represents a related metabolic pathway. The colour and size of each dot denote the −ln(p) value and pathway impact value, respectively.


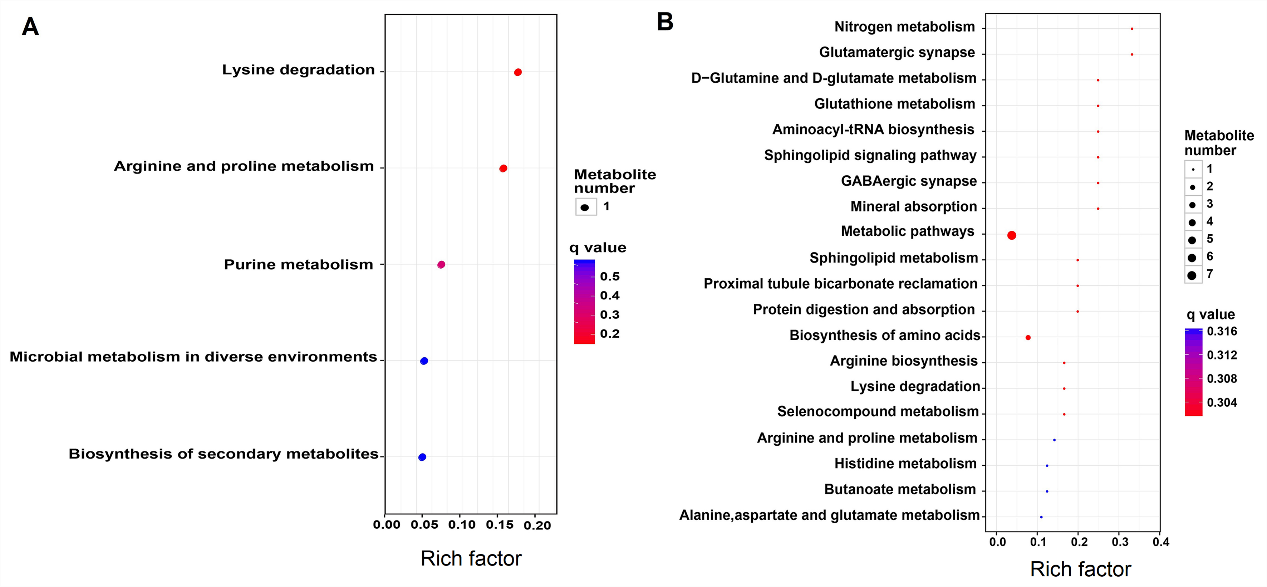


Supplementary Figure 4. Bubble plot of pathway analysis. (A) The pathways that significantly differed between the L-before and H-before groups. (B) The pathways that significantly differed between the L-after and H-after groups. Each dot represents a related metabolic pathway. The colour and size of each dot denote the−ln(p) value and pathway impact value, respectively.
